# Effect of oral probiotics on clinical efficacy and intestinal flora in elderly severe pneumonia patients

**DOI:** 10.1097/MD.0000000000036320

**Published:** 2023-12-01

**Authors:** Yonglin Zhu, Guannan Ma, Wei Ren, Zhenyu Hu, Ling Zhou, Xin Zhang, Na Zhao, Mingding Zhang, Lei Yan, Qian Yu, Xuetong Liu, Jichao Chen

**Affiliations:** a Aerospace Center Hospital, Beijing, China; b Key Laboratory of Digital Technology in Medical Diagnostics of Zhejiang Province, Hangzhou, China; c Beijing D.A. Medical Laboratory, Beijing, China.

**Keywords:** elderly patients, gut microbiota, probiotics, severe pneumonia

## Abstract

Complex microbial ecosystems in both gastrointestinal and respiratory systems have been found to have a significant impact on human health. Growing evidence has demonstrated that intestinal dysbiosis can increase vulnerability to pulmonary infections. However, changes in the composition and activity of the intestinal flora after probiotic supplementation may alter the disease state of the host. The effects of probiotics on the improvement of diseases, such as severe pneumonia (SP), in intensive care units (ICUs) remain controversial. We retrospectively included 88 patients diagnosed with severe pneumonia between April 2021 and June 2022. The patients were divided into 2 groups: a probiotic group (n = 40) and a control group (n = 48). In addition, changes in CRP, PCT, WBC, IL-6, Clostridium difficile toxin, and PSI pneumonia scores were assessed. Changes in the gut microbiome of the patients were assessed using amplicon sequencing. Compared to the control group, a significant reduction in the incidence of length of hospital stay was observed in the probiotic group, but there were no significant differences in the mortality rate, duration of fever, diarrhea, and constipation. After probiotic treatment, CRP, PCT, WBC, and PSI score were significantly lower than before, and better clinical efficacy was achieved in the probiotic group for the duration of antibiotic therapy. Gut microbiota analysis revealed that the abundance of opportunistic pathogens (e.g., *Massilia*) increased remarkably at the genus level in the control group, and a significant increase in *Erysipelotrichaceae_ge* was observed after probiotic intervention. The control group showed an increase in opportunistic pathogens (*Citrobacter, Massilia*) during the antibiotic treatment. Probiotics interventions inhibit the growth of opportunistic pathogens. In addition, we found that the population of butyrate-producing bacteria (e.g., *Ruminococcaceae UCG-005*) increased following probiotic treatment.

## 1. Introduction

Severe Pneumonia (SP) is a serious threat to human health. The global health problem caused by severe pneumonia speeds up the development and progression of cancer, diabetes, AIDS, and other diseases, and is the leading cause of death worldwide compared to other infectious diseases.^[1–3]^ In particular, severe pneumonia results in the utilization of substantial healthcare resources and economic burden, particularly during ICUs hospitalization.^[4,5]^ A study by Cavallazzi et al showed that 23% of patients admitted to the ICU with severe community-acquired pneumonia had a 30-day mortality rate of 27% and a 1-year mortality rate of 47%. Hospital-acquired pneumonia is a common nosocomial infection, with a mortality rate of 13% to 28%.^[6]^

The lung, as an organ, is considered sterile, and the Human Microbiome Project in the United States did not include the lung microbiome in its study.^[7]^ However, in 2010, a research group from Imperial College London examined the lung microbiome of asthma patients and confirmed that the lower airways were not sterile for the first time.^[8]^ This discovery has influenced the understanding of microorganisms in respiratory immunity and the mechanism of lung disease,^[9]^ and provides new ideas for the treatment of lung diseases, especially chronic obstructive pulmonary disease and bronchial asthma.^[10]^ Consequently, its believed that the gut microbiota and the lung microbiota are connected through potential anatomical pathways, mutual interference between microbiota and immune response, and play several important roles in the development, regulation, and therapy of respiratory diseases. This association is referred to as the gut-lung axis.^[11–13]^ A healthy gut microbiota protects against severe pneumonia and regulates innate and adaptive immune responses to defend against lower respiratory tract pathogens.^[14]^ Some researchers have reported that gut microbiota may play a protective role in bacterial and viral lung infections through immune regulation.^[15–17]^ A growing body of research has revealed that probiotics, such as Lactobacillus rhamnosus, can improve antiviral defenses, reduce pro-inflammatory cytokines in systemic and respiratory infections, and help improve intestinal and lung barriers and homeostasis.^[18,19]^ Several clinical trials have also shown that oral intestinal probiotics can benefit patients with COVID-19 pneumonia.^[20,21]^ Given that increasing knowledge of microbial dysbiosis in the pneumonia and how to explore the potential therapeutic through 16S rRNA gene sequencing, the aim of this study is to conduct a prospective study in patients with pneumonia in the respiratory and intensive care unit to investigate the clinical value of probiotic treatment in pneumonia and its effect on gut flora, and to provide new ideas for clinical practice.

## 2. Materials and methods

### 2.1. Patients and groups

This was a retrospective case-control study involving 88 patients with severe pneumonia treated in the Department of Respiratory and Critical Care Medicine at the China Aerospace Center Hospital from April 2021 to June 2022. There were 59 males and 29 females, aged 55 to 90 years, with an average age of 81.8 (55–90) years. The patients were randomly divided into the probiotic and control groups. There were 40 patients in the probiotic group(29 males and 11 females), with an average age of 81 (55–90) years. There were 48 patients in the control group, including 30 males and 18 females, with an average age of 82.5 (55–90) years. There was no significant difference (*P* > .05) in sex, age, or other general data between the 2 groups, which were comparable. However, as this was a non-randomized study, there are many potential sources of bias. This study was approved by the ethics committee of our hospital. All patients and their relatives were informed of the study and signed an informed consent form. Our study size was determined by including all data available within the study period on the topic of interest.

We followed the ethical standards of the Declaration of Helsinki throughout the study. The data were acquired retrospectively by the Department of Respiratory and Critical Care Medicine of Aerospace Center Hospital. The data were collected between April 2021 to June 2022. A total of 80 patients with a final diagnosis of severe pneumonia were included.

Patients in both groups were treated according to the guidelines,^[22,23]^ including active anti-infection, correction of water and electrolyte disorders, low-flow oxygen inhalation, anti-shock, anticoagulant therapy, sedation, nutritional support, cardiac strengthening, expectorant and other comprehensive medical treatments. Mechanical ventilation, prevention and treatment of DIC were administered according to the patient condition. Patients in both groups were provided reasonable guidance on the control diet, and patients in both groups received professional and standardized nursing.

The probiotic group was treated with live combined Bacillus subtilis and Enterococcus faecium enteric-coated capsule (Beijing Hanmei Pharmaceutical Co., LTD., Approval number: S20030087, 0.5 g, 3 times/day) and Bacillus Licheniformis capsule (Northeast Pharmaceutical Group Shenyang First Pharmaceutical Co., LTD., Approval number:10950019, 0.5 g, 3 times/day). In cases of oral difficulty, the patients were fed through anasointestinal tube after dissolving 25 ml of sterilized water.

### 2.2. Clinical detection and intestinal Amplicon Library sequencing

Clinical outcomes, length of hospital stay, and the number of days of antibiotic use were collected. Each patient was monitored for gastrointestinal symptoms (diarrhea or constipation), duration and peak of fever, presence or absence of sepsis, presence or absence of mechanical ventilation, and presence or absence of deep venous catheterization. Patients were assessed for PSI pneumonia scores before and 21 days after treatment. The laboratory test results of the patients were collected, including CRP, PCT, WBC, IL-6 and Clostridium difficile toxin test results.

Fecal samples were collected from some patients before (pre-probiotic) and after (post-probiotic) probiotic intervention. Fecal bacterial DNA was extracted using TIANamp Stool DNA Kit (Invitrogen, California, USA) according to the manufacturer protocol, DNA concentration and integrity were determined using a Nanodrop ND 1000 Spectrophotometer (Thermo Fisher Scientific, United States). PCR amplification of the bacterial 16S rRNA gene V3–V4 region was performed using the forward primer 341F (5’-CCTACGGGNGGCWGCAG-3’) and the reverse primer 785R (5’-GACTACHVGGGTATCTAATCC-3’). Sequencing libraries were prepared using Illumina TruSeq Nano DNA LT Library Prep Kit (Illumina, Delaware, USA), and sequenced on an Illumina Hiseq2500 platform. The sequencing service was provided by Dian Diagnostics Group Co Ltd. (Hangzhou, China).

### 2.3. Bioinformatic analysis

The 16S rRNA gene sequence data sets were initially processed by merging and demultiplexing into each sample using QIIME (version 1.9.0) with default parameters. Subsequently, USEARCH software, employing the UCHIME algorithm, was utilized to identify and eliminate chimera sequences. An open-reference operational taxonomic unit selection was then conducted using USEARCH (version 7) against the Greengenes database (version 13.8) with a sequence similarity threshold of 97%. This operational taxonomic unit pick was employed for subsequent analysis of microbiota composition. Alpha diversity was determined using QIIME software, employing a sequence similarity threshold of 97%. Sequence coverage was evaluated in mothur using rarefaction curves and Good coverage. Beta diversity was assessed by employing unweighted and weighted UniFrac distances, which were calculated by sub-sampling ten times using QIIME. The resulting distances were visualized using NMDS with a distinct algorithm. Hierarchical clustering was conducted and a heatmap was generated using Spearman rank correlation coefficient as a distance measure. This analysis was performed using a customized script developed using the R statistical package. To identify the microorganismal features that distinguish the gut microbiota, the Metastats tool was employed.

### 2.4. Statistical analysis

IBM SPSS 26.0 statistical software was used for data processing and analysis. Measurement data are expressed as mean ± standard deviation. The chi-square test was used to calculate *P* values for qualitative data. The quantitative data conforming to normal distribution were analyzed using an independent sample t-test, and *P* < .05 was considered statistically significant. Data that did not meet the normal distribution were tested by rank sum test, and the data were divided into P< 0.05, which was considered statistically significant. Repeated measures analysis of variance was used for data with grouping variables and time variables, with *P* < .05, considered statistically significant.

## 3. Result

### 3.1. Baseline demographics and clinical informations

In this study, there was no significant difference in sex (Male (%) in Probiotic: 72.50%, Male (%) in Control: 27.50%; Female (%)in Probiotic: 62.50%, Female (%) in Control: 37.50%; *P* = .340) and age (Probiotic: 81 (55–90), Control:82.5 (55–90); *P* = .195) between the 2 groups. There was no significant difference between the 2 groups in the pneumonia PSI score before the treatment (Probiotic: 195.5 (134–273), Control: 208 (145–300); *P* = .648). There were no significant differences in the laboratory test results before treatment, such as C-reactive protein (CRP), procalcitonin (PCT), interleukin-6 (IL-6), white blood cell count (WBC), positive rate of Clostridium difficile toxin test, and PSI score (see Table 1). Enrollment procedures were performed.

**Table 1:** Basic information and clinical features of patients.

		Probiotic group (n = 40)	Control group (n = 48)	*P* value
Male (%)		72.50%	62.50%	.340
Mean age		81 (55–90)	82.5 (55–90)	.195
Before the treatment	Mean PSI score	195.5 (134–273)	208 (145–300)	.648
	CRP (mg/L)	99.0 (13.8–150.0)	70.79 (10.9–150)	.383
	PCT (ng/mL)	1.05 (0.1–21.6)	1.05 (0.1–17)	.704
	WBC(×10^9/L)	10.32 (2.70–22.34)	9.95 (2.70–21.93)	.578
	IL-6 (pg/mL)	97.4 (9.8–4000.0)	117.7 (12–3815.7)	.700
	Clostridium difficile toxin positive rate (%)	2.5%	6.2%	.623
After the treatment	Mean PSI score	102.5 (0–273)	178 (0–300)	.030*
	CRP (mg/L)	45.15 (7.8–150.0)	50.80 (5–150.0)	.381
	PCT (ng/mL)	0.50 (0.1–13.1)	0.60 (0.1–20.3)	.425
	WBC(×10^9/L)	7.61 (1.98–19.24)	7.72 (1.98–57.22)	.485
	IL-6 (pg/mL)	47.45 (9.4–4000)	60.7 (6.8–4000)	.51
	Clostridium difficile toxin Positive rate (%)	20%	31.2%	.232
Days of fever^a^		11 (2–19)	10.5 (0–30)	.434
Peak of body temperature (°C)		38.6 (37.8–39.7)	38.25 (37.1–39.9)	.089
Length of stay		20.5 (14–27)	25 (10–41)	.045*
Days of antibiotic use		12 (8–31)	13 (6–30)	.069
Incidence of constipation (%)^b^		12.50%	23%	.207
Incidence of diarrhea (%)^c^		35%	50%	.157
Incidence of sepsis (%)		22.5%	27.0%	.476
Invasive mechanical ventilation (%)		45.0%	43.7%	.906
Central vein (%)		40.0%	35.40%	.658
Mortality rate (%)		20.0%	29.2%	.459

Categorical variables were presented as percentages, and the χ^2^ test was used to compare the differences between the 2 groups. Continuous variables were presented in the “median (Min - Max)” format.

* *P* < .05.

CRP = C-reactive protein, IL-6 = interleukin-6, PCT = procalcitonin, PSI = Pneumonia Severity Index, WBC = White Blood Cells.

a Fever was defined as axillary temperature ≥ 37.3°C.

b Constipation was defined as defecation <3 times per week, difficult defecation, dry stool or the need for manual assistance.

c Diarrhea was defined as the number of bowel movements ≥ 3 per day, thin stool, increased moisture, daily bowel movements exceeding 200g, or containing undigested food or pus, blood, and mucus.

### 3.2. Clinical outcome and characteristics

**Figure 1.**
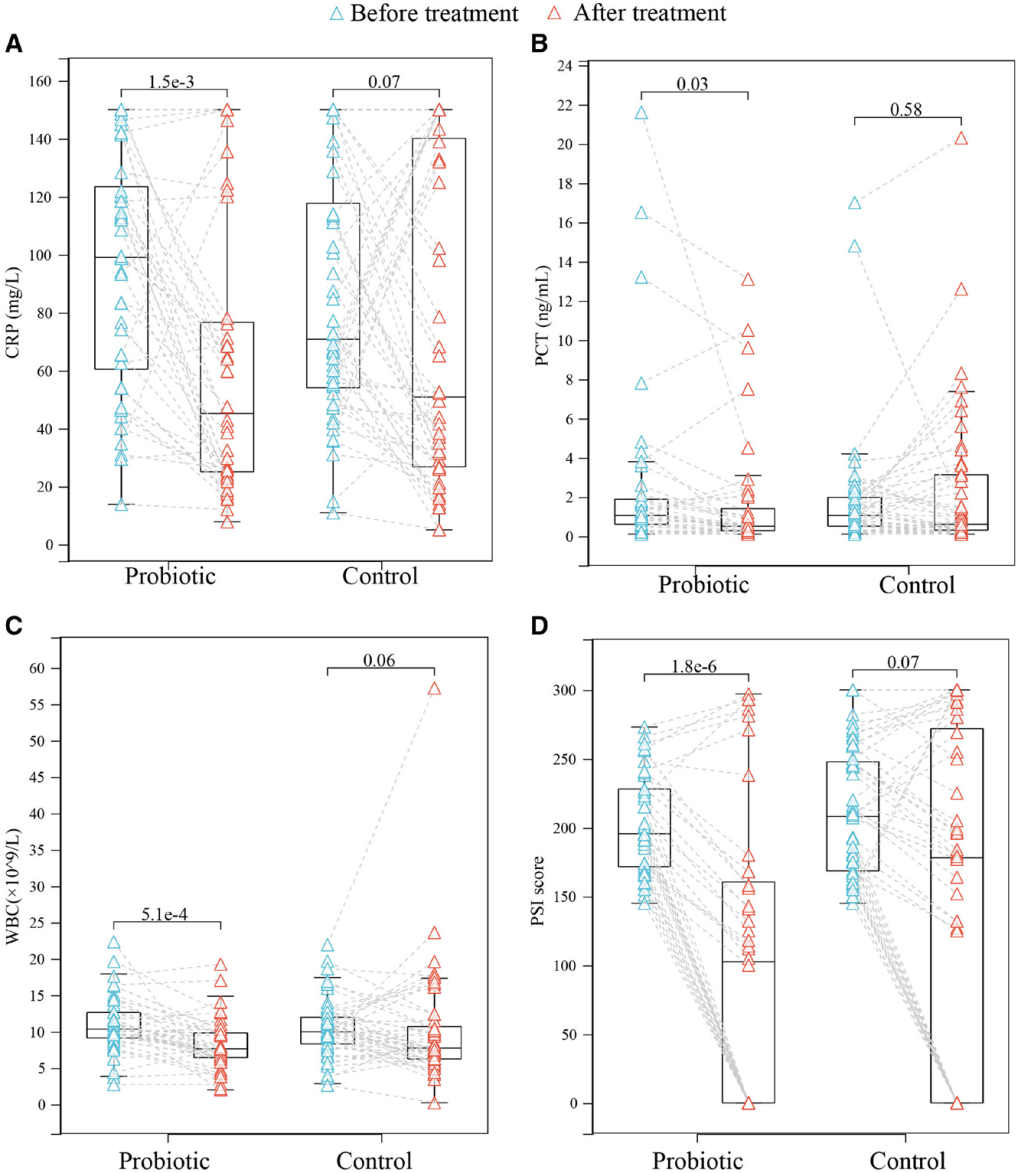
Clinical indexes with significant differences before and after treatment.

We analyzed the clinical outcomes and indices of the patients. There were no significant differences in the number of days of fever (Probiotic:11 (2–19), Control:10.5 (0–30); *P* = .434), peak body temperature(Probiotic:38.6 (37.8–39.7), Control:38.25 (37.1–39.9); *P* = .089), Length of stay (Probiotic:20.5 (14–27), Control:25 (10–41); *P* = .045), incidence of sepsis (Probiotic:22.5%, Control:27.0%; *P* = .476), mortality (Probiotic:20%, Control:29.2%%; *P* = .459), invasive mechanical ventilation rate (Probiotic:45%, Control:43.7%%; *P* = .906), and central venous catheterization rate (Probiotic:40%, Control:40%; *P* = 1) between the Probiotic and Control groups. There was no significant difference in CRP (Probiotic:45.15 (7.8–150), Control:50.80 (5–150.0); *P* = .381), PCT (Probiotic:0.50 (0.1–13.1), Control:0.60 (0.1–20.3); *P* = .425), IL-6 (Probiotic:47.45 (9.4–4000), Control:60.7 (6.8–4000); *P* = .51) and WBC (Probiotic:7.61 (1.98–19.24), Control:7.72 (1.98–57.22); *P* = .485) between the Probiotic and the Control group. However, after 21 days of treatment, CRP, PCT, WBC, and PSI scores of the probiotic group were significantly lower than those at the time of enrollment (Fig. 1A–D).

### 3.3. Fecal microbiota analysis

#### 3.3.1. Diversity of fecal microbiota.

**Figure 2.**
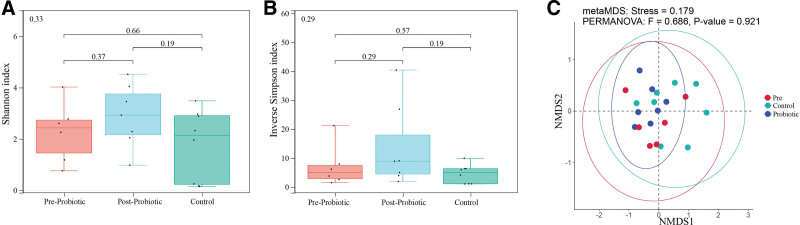
Diversity of the fecal microbiota after probiotic intervention for patients. Diversity indices, such as Shannon index (A), Inversed Simpson index (B), and Bray-Curtis NMDS (C) were used to evaluate the overall structure of the fecal microbiota after probiotic therapy. The data are presented as the mean ± standard deviation. Unpaired t-tests (two-tailed) were used to analyze variation among the fecal microbiota.

By analyzing the diversity of fecal microbiota, we found no significant difference in α and β diversity among Control, pre-probiotic and post-probiotic groups. The Shannon index and inverse Simpson index did not change significantly with probiotic administration, and there was no significant difference in the Bray-Curtis NMDS analysis of β diversity (Fig. 2).

#### 3.3.2. Microbial community structure.

**Figure 3.**
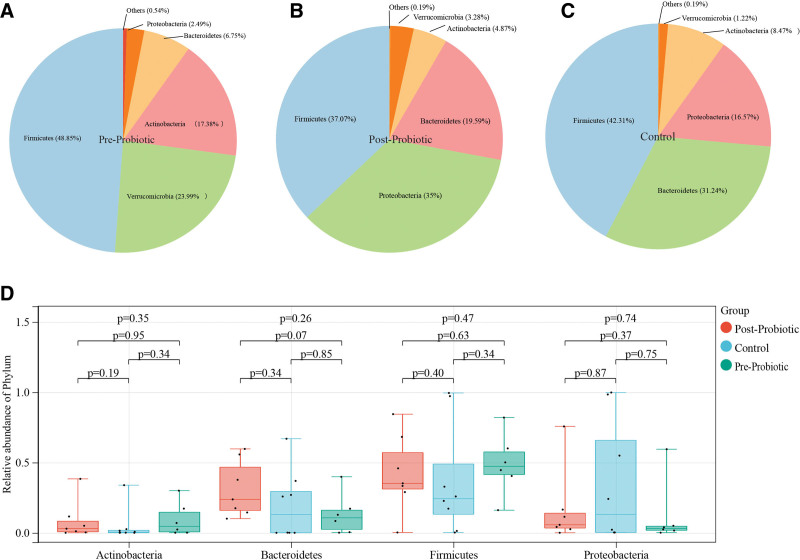
Different bacterial taxa among control and pre-probiotic and post-probiotic treatment groups. (A–C) Microbiome distribution at the phylum level in control and pre-probiotic and post-probiotic treatment groups D. Comparison of the relative abundance of bacterial taxa at phylum level.

In this study, most sequences of the fecal microbiota were grouped into 4 phylums, including Firmicutes, Bacteroidetes, Proteobacteria, and Actinobacteria (Fig. 3A, 3B, and 3C). Compared with the Control group, there were no differences in the fecal flora of the 4 phylums in the pre-probiotic and post-probiotic groups (Fig. 3D, Table S1, http://links.lww.com/MD/K837).

**Figure 4.**
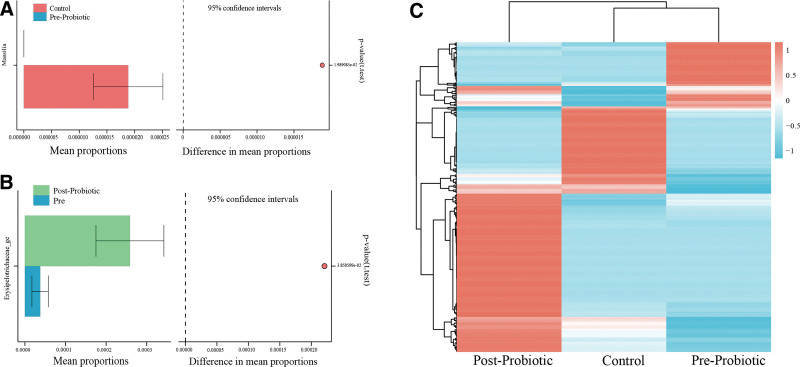
Different bacterial taxa among control and pre-probiotic and post-probiotic treatment groups. (A) STAMP analysis among control and pre-probiotic groups. (B) STAMP analysis among pre-probiotic and post-probiotic treatment groups. (C) PLS-DA of control and pre-probiotic and post-probiotic treatment groups.

**Figure 5.**
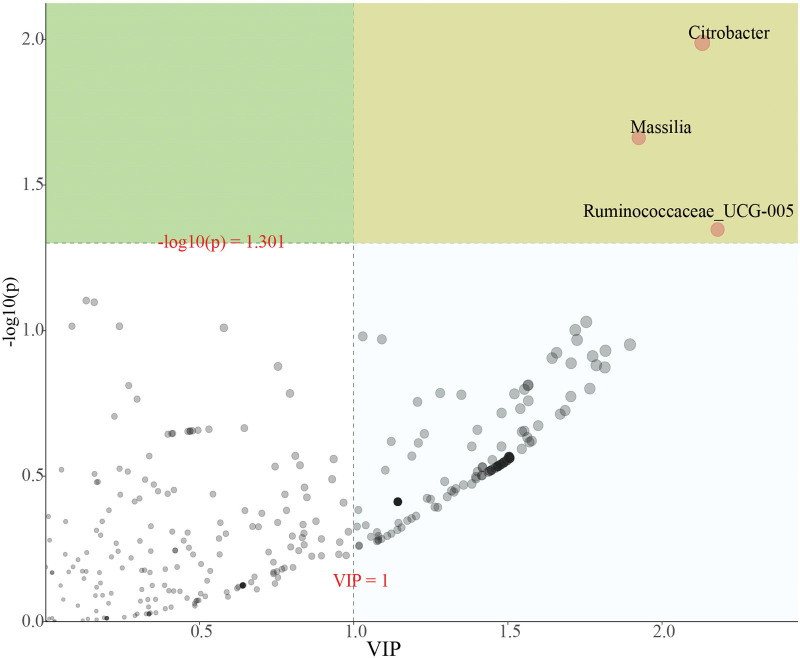
Heatmap of the key genera in the fecal microbiota among control and pre-probiotic and post-probiotic treatment groups. The color of the spots in the panel represents the relative abundance of the genus in each sample. The relative abundance of the bacteria in each genus is indicated by a gradient of color from blue (low abundance) to red (high abundance).

At the genus level, opportunistic pathogens (e.g., *Massilia*) were significantly increased in the Control group (Fig. 4A), and *Erysipelotrichaceae_ge* was significantly increased before and after the post-probiotic intervention (Fig. 4B). Figure 4C shows the heatmap of bacterial genus in the pre-probiotic and post-probiotic treatment groups, as well as in the Control group, which represents the relative percentage of the most abundant genus identified in each sample, and there were obvious differences between the control, pre-probiotic and post-probiotic treatment groups. Significant differences in fecal microbiota between the 3 groups were compared using PLS-DA, consistent with previous analysis. With antibiotic treatment, opportunistic pathogens (*Citrobacter, Massilia*) increased in the control group, but the increase in opportunistic pathogens was inhibited by probiotics intervention. Butyrate producing bacteria (such as *Ruminococcaceae_UCG-005*) increased after probiotic treatment (Fig. 5, Table S2, http://links.lww.com/MD/K838). In conclusion, the diversity of fecal microbiota in patients with severe pneumonia showed a trend toward microbiota recovery after short-term probiotic treatment, but there was no marked improvement in fecal microbiota composition.

## 4. Discussion

Severe pneumonia is common in the elderly population. The clinical symptoms are not particularly obvious, and they may be similar to other diseases, such as cough, expectoration, and fever. It is mainly caused by extrapulmonary symptoms, which have a serious impact on the quality of life of the elderly.^[24]^ Elderly individuals often have a variety of chronic complications. To prevent pulmonary infection, it is necessary to perform chest X-ray examination in time and conduct repeated sputum cultures to achieve early prevention and treatment.^[25]^ In clinical practice, the type of pneumonia is infectious, and the source of pathogens is mainly contact with the population, including pneumococcus, gram-negative bacilli and atypical pathogens. Therefore, it is very important to select appropriate antibacterial drugs in the treatment of severe pneumonia in elderly patients.^[26]^ Elderly patients have decreased immune function, poor body resistance, and a gradually disordered intestinal flora. They are easily affected by external factors that lead to dysbacteriosis and diarrhea symptoms.^[24]^ In the treatment of severe pneumonia in the elderly, due to the unclear pathogenic bacteria, patients are treated with a large number of broad-spectrum antibiotics. The treatment cycle of antibiotics is long and the dosage is large, which can easily cause intestinal flora imbalance, destroy intestinal microecology in the body, produce diarrhea symptoms, and also aggravate the condition of elderly patients with severe pneumonia.^[27]^ In recent years, microecological agents such as *Bifidobacterium, Clostridium butyricum, Streptococcus faecalis, Lactobacillus*, and *Bacillus* have been used in clinical practice to treat elderly patients with severe pneumonia and prevent the occurrence of antibiotic-related diarrhea symptoms.^[28]^ Live combined Bacillus subtilis and Enterococcus faecium enteric-coated capsule is a commonly used intestinal probiotic preparation. It contains *Bacillus subtilis* and *Enterococcus faecium*, which are members of the normal flora in the gut of healthy people. Bacillus Licheniformis capsule is a live preparation of *Bacillus licheniformis*. After oral administration, it can rapidly grow and multiply in the intestinal tract, resulting in a hypoxic environment. It can promote the growth and reproduction of healthy anaerobic bacteria, such as *Bifidobacterium, Lactobacillus, Bacteroides*, and *Petostreptococcus*, and pathogenic bacteria, such as *Staphylococcus* and *Candida albicans*. Several randomized controlled trials have reported that probiotics may reduce the incidence of ventilator associated pneumonia.^[29]^ Additionally, it has been observed that treatment with probiotics and prebiotics can regulate the levels of various cytokines, such as IL-4, 5, 13, 25, 33, as well as leukotrienes. Furthermore, it has been found that these interventions can also modulate the gene expression of AKT, NLR3, NF-κB, MyD88, and MUC5a, as well as control peribronchial inflammation and PI3K gene expression.^[30]^

This retrospective study found that the imbalance of gut microbiota in elderly patients with severe illness can be effectively improved by the combined use of live combined Bacillus subtilis and Enterococcus faecium enteric-coated capsule. The probiotics play a protective role in the intestines and stomach of patients, indirectly improve the body damage caused by the use of antibiotics, and resist the invasion of viruses and bacteria, and enhance the immune function of patients. The combined use of live combined Bacillus subtilis and Enterococcus faecium enteric-coated capsule and Bacillus Licheniformis capsule can improve the efficacy of treatment in elderly patients with severe pneumonia, indicating that by taking probiotics, the intestinal environment of patients is improved, and the treatment effect of pneumonia is indirectly improved. In conclusion, probiotics can alter the intestinal microecological environment of elderly patients with severe pneumonia, improve clinical symptoms, reduce the damage caused by antibiotics, and improve the quality of life of patients.

The results of this study showed that the application of exogenous intestinal probiotics in the treatment of pneumonia had some beneficial effects, especially the important value was observed in reducing the gastrointestinal burden and the antibiotic use cycle. However, due to the limited factors of this study, such as insufficient sample size, complex types of pathogens, the mechanism of the “lung-gut axis” has not been fully elucidated, and there is a complex relationship between the host and the microbiota. Further studies are needed to verify our findings. Therefore, the generalizability of the results is limited.

## 5. Conclusion

This study showed that intestinal probiotic supplementation plays an important role in the treatment of elderly patients with severe pneumonia, which indicated that intestinal probiotics supplementation can improve the gut microbiome, clinical symptoms and quality of life in elderly patients with severe pneumonia.

## Acknowledgments

The authors are grateful to the patients for their participation and to Dr Yumin Wang of the Aerospace Center Hospital for her guidance and assistance.

## Author contributions

**Conceptualization:** Jichao Chen.

**Data curation:** Zhenyu Hu, Ling Zhou, Na Zhao.

**Formal analysis:** Guannan Ma.

**Investigation:** Yonglin Zhu, Wei Ren, Ling Zhou, Xin Zhang, Na Zhao, Mingding Zhang, Lei Yan.

**Methodology:** Yonglin Zhu, Wei Ren, Xin Zhang.

**Resources:** Yonglin Zhu.

**Software:** Zhenyu Hu, Ling Zhou.

**Supervision:** Xuetong Liu.

**Visualization:** Guannan Ma.

**Writing – original draft:** Yonglin Zhu, Guannan Ma, Wei Ren, Qian Yu, Jichao Chen.

**Writing – review & editing:** Yonglin Zhu, Guannan Ma, Wei Ren, Qian Yu, Xuetong Liu, Jichao Chen.

## Supplementary Material




